# Chitosan Film Containing *Mansoa hirsuta* Fraction for Wound Healing

**DOI:** 10.3390/pharmaceutics12060484

**Published:** 2020-05-27

**Authors:** Joquebede Rodrigues Pereira, Gabriela Suassuna Bezerra, Allanny Alves Furtado, Thaís Gomes de Carvalho, Valéria Costa da Silva, Amanda Lins Bispo Monteiro, Gerlane Coelho Bernardo Guerra, Raimundo Fernandes de Araújo Júnior, Antônio Euzébio Goulart Sant’Ana, Matheus de Freitas Fernandes-Pedrosa, Daniel de Melo Silva, Eduardo Pereira de Azevedo, Tania Maria Sarmento Silva, Telma Maria Araújo Moura Lemos, Ádley Antonini Neves de Lima

**Affiliations:** 1 Department of Clinical and Toxicological Analysis, College of Pharmacy, Federal University of Rio Grande do Norte, Natal, RN 59012-570, Brazil; joque.rodrigues@gmail.com (J.R.P.); telmaml@gmail.com (T.M.A.M.L.); 2 Department of Pharmacy, Center of Health Sciences, Federal University of Rio Grande do Norte, Rio Grande do Norte, RN 59012-570, Brazil; gabisuassuna.08@gmail.com (G.S.B.); allannyfurtado08@gmail.com (A.A.F.); mffpedrosa@gmail.com (M.d.F.F.-P.); 3 Department of Morphology, Federal University of Rio Grande do Norte, Natal, RN 59072-970, Brazil; thaisbida2013@gmail.com (T.G.d.C.); araujojr@cb.ufrn.br (R.F.d.A.J.); 4 Department of Biophysics and Pharmacology, Biosciences Center, Federal University of Rio Grande do Norte, Natal 59072-970, Brazil; vcs.biomed@gmail.com (V.C.d.S.); gerlaneguerra@gmail.com (G.C.B.G.); 5 Department of Chemistry, Universidade Federal Rural de Pernambuco, Recife-PE 52171-900, Brazil; amandalinsbmonteiro@gmail.com (A.L.B.M.); sarmentosilva@gmail.com (T.M.S.S.); 6 Institute of Chemistry and Biotechnology, Federal University of Alagoas, Campus A.C. Simões, Maceió, AL 57072-970, Brazil; aegsal@gmail.com; 7 Department of Chemistry and Exact Sciences, State University of Southwest of Bahia, Jequié, BA 45208-091, Brazil; daniel.melo@uesb.edu.br; 8 Graduate Program of Biotechnology, Laureate International Universities-Universidade Potiguar (UnP), Natal 59056-000, Brazil; azevedoep@hotmail.com

**Keywords:** chitosan, *Mansoa hirsuta*, films, wound healing, biomaterial

## Abstract

Chitosan films entrapped with the *Mansoa hirsuta* fraction (CMHF) was developed as a new dressing for wound care. The chromatographic profile of the *M. hirsuta* fraction (MHF) was evaluated by ultra-high-performance liquid chromatography-quadrupole time-of-flight mass spectrometry, and the results showed that MHF is rich in acid triterpenes. Physicochemical characterization of the films prepared using the solvent casting method was performed by Fourier transform infrared spectroscopy (FTIR), X-ray diffraction (XRD), thermogravimetry (TGA), differential scanning calorimetry (DCS), scanning electron microscopy (SEM), atomic force microscopy (AFM), and mechanical properties. CMHF exhibited characteristic bands of both chitosan and MHF, revealing a physical mixture of both. CMHF presented an amorphous nature, thermostability, and dispersion of MHF in the chitosan matrix, resulting in a rough structure. Incorporation of *M. hirsuta* fraction into chitosan matrix favorably enhanced the mechanical performance and films thickness. The in vivo wound treatment with CMHF for seven days showed a characteristic area of advanced healing, re-epithelization, cell proliferation, and collagen formation. Furthermore, wound closure reached 100% contraction after 10 days of treatment with modulation of interleukins. The incorporation of *M. hirsuta* fraction into chitosan films was advantageous and showed great potential for stimulating wound repair and regeneration.

## 1. Introduction

Wounds are caused by genetic diseases or traumatic injuries that result in the disruption of normal tissue structure and function, which can cause irreparable damage that usually results in disability and death [1,2]. Wound healing is an essential process for restoring tissue integrity and function. Therefore, the demand for an efficient treatment that is able to shorten healing time and reduce the risk of unwanted complications is increasingly high [3]. In recent years, major efforts have been made to develop new therapeutic alternatives that are more appropriate for restoring normal skin architecture after wound damage [4]. Considering the local wound as the main target for therapeutic strategies, polymeric films represent a promising approach for the treatment of these injuries since they produce a more prolonged effect, are easily and conveniently applied, and are in direct contact with the wound [5].

Chitosan is a natural polymer that has become an important alternative for developing novel wound dressings due to its easy processing, biocompatibility, biodegradability, non-toxicity, antimicrobial properties, and resemblance to extracellular matrix [6]. Obtained through deacetylation of chitin, chitosan is composed of beta-1,4-glycosidic bonded D-glucosamine and N-acetyl D-glucosamine monomers [7,8]. Chitosan is a biomaterial that favors multi-stage wound healing by promoting the proliferation and activation of inflammatory cells toward the granulation tissue and by accelerating rapid dermal regeneration and re-epithelization [9]. It has the ability to absorb large amounts of fluids and, therefore, keeps the wound environment moisturized, which is an essential feature for any dressing material [10]. In addition, chitosan has high film-forming capacity and mucoadhesive property [11].

Thus, due to its ability to adhere to the epithelial surface and prolong the contact time and drug release, the incorporation of therapeutic agents into polymeric dressings has become a promising approach to better control the inflammatory process as well as prevent infections and stimulate tissue regeneration [12]. In this context, natural products such as the ones obtained from the *Mansoa hirsuta*, with their antioxidant and anti-inflammatory properties, are promising candidates for incorporation into polymeric matrices for a faster and more effective wound care [13,14].

*Mansoa hirsuta* D.C., known as cipó-de-alho (Brazil), is a Bignoniaceae plant endemic of the semiarid Brazilian region [15]. In traditional medicine, the leaves of this species have been used to treat diabetes and sore throats [16,17]. The raw ethanol extract and fractions from *M. hirsuta* contains many active components que have a wide spectrum of biological and pharmacological activities. Such components include phenols, tannins, steroids, triterpenes, saponins, flavonols, flavanonols, flavanones, xanthones, anthocyanins, anthocyanidins, and flavonoids [18]. Some biological activities have been reported for this species, which include inhibition of tumor necrosis factor alpha (TNF-α) and cyclooxygenase-1 [19,20] as well as anti-hypertensive [21], antifungal [22], and antioxidant activities [18]. Therefore, *M. hirsuta* represents a potential source of phytochemicals against inflammatory and others pathologies [18].

A previous study demonstrated the immunomodulatory potential of this species [23]. Moreover, the fraction of *M. hirsuta* leaves (MHF) was fractioned and purified by column chromatography in silica gel, obtaining the ursolic and oleanolic acids, which were also effective in reducing lymphocyte proliferation and the formation of nitric oxide by macrophages [23].

So far as is known, there are no studies on MHF loaded into chitosan films. Thus, the aim of this work was to analyze the chemical composition of MHF as well as to develop and characterize novel chitosan/*M. hirsuta* fraction films as a potential dressing for wound repair. The characterization of MHF was performed by ultra-high performance liquid chromatography-quadrupole time-of-flight mass spectrometry (UPLC-QTOF-MS / MS) and the films were prepared using the solvent casting method and characterized by Fourier transform infrared spectroscopy (FTIR), X-ray diffraction (XRD), thermogravimetry (TGA), differential scanning calorimetry (DSC), scanning electron microscopy (SEM), and atomic force microscopy (AFM) to validate its applicability. In addition, the mechanical and swelling properties, as well as the wound healing efficacy, were investigated.

## 2. Materials and Methods

### 2.1. Material

Chitosan (molecular weight of 190.000-310.000 kDa and deacetylation degree of 75–85%) was obtained from Sigma-Aldrich (St. Louis, MO, USA). Ketamine hydrochloride and xylazine hydrochloride were obtained from Syntec (Santana de Parnaíba, SP, Brazil). All other reagents and solvents were of analytical grade.

### 2.2. Vegetal Material

*M. hirsuta* was collected by Teonildes Nunes in Santo Inácio, Bahia, Brazil, (11°19′S, 42°40′W) where one specimen was deposited at the Herbarium of Feira de Santana State University (registration # 59456). Partition of the crude ethanolic extract (250g) from *M. hirsuta* leaves gave the acetate phase (40g), which was funnel filtered with silica resulting in 15 g of the MHF. This process was performed by Daniel de Melo Silva [23] at the Natural Resources Research Laboratory (UFAL-AL). This research was authorized by the National Genetic Heritage Management System and Associated Traditional Knowledge (SISGEN) registration No. A350944 and performed according to the Brazilian Biodiversity Law (Federal Law No. 13.123/2015).

### 2.3. Characterization of MHF UPLC-QTOF-MS/MS

The chromatographic separation of compounds was performed on the ACQUITY UPLC with a conditioned autosampler at 4 °C using the Waters Acquity UPLC BEH C18 column (2.1 mm × 50 mm, 1.7 μm) (Waters, Milford, MA, USA). The mobile phase consisting of water with 0.1% formic acid (solvent A) and acetonitrile with 0.1% formic acid (solvent B) was pumped at a flow rate of 0.4 mL min^−1^. Gradient elution was applied starting from 40% B: 0–8 min, 40–85% B; 8-9 min, 85–95% B. The injection volume was 5–8 μL. The MS analysis was performed on a Xevo G2 QTOF (Waters MS Technologies, Manchester, UK), a quadrupole time-of-flight tandem mass spectrometer coupled with an electrospray ionization source in positive and negative ion mode. The scan range was from 50 *m/z* to 1200 *m/z* for data acquisition.

In addition, MS/MS spectra (MS^E^) experiments were carried out, which allow both precursor and product ion data to be acquired in one injection. The source conditions were as follows: 3.5 kV capillary voltage; 120 °C source temperature; 450 °C desolvation temperature; 100 Lh^−1^ cone gas flow rate; 800 Lh^−1^ desolvation gas (N_2_) flow rate and 30 V cone voltage. Leucine-enkephalin (500 pg.mL^−1^) was used as a standard/reference compound to calibrate the mass spectrometer during the analyses. MS analyses were initially performed in both the negative and positive ionization modes, but the latter was preferred as it gave more structural information. All data acquisition and analyses were controlled using the Waters MassLynx v 4.1 software. To obtain abundant fragmentation ions, several values of collision energy (6–40 V) were selected.

### 2.4. Preparation of Chitosan/M. hirsuta Fraction Films

The blank chitosan and chitosan/*M. hirsuta* fraction films were prepared using the solvent casting method as previously described with some modifications [24]. Chitosan 1% (*w/v*) solution was obtained by dissolving the polymer in acetic acid (1% *v/v*) under magnetic stirring (24.000 rpm) for 24 h at room temperature. For preparation of blank chitosan films (BFs), the polymeric solution was poured in Petri dishes (47 mm in diameter) followed by oven drying at 40 °C overnight. For the production of chitosan films entrapped with *M. hirsuta* fraction (CHMF), MHF at a concentration of 1.5% (*w/w*) was first dissolved in ethyl alcohol, and 2 mL of this solution was mixed with 8 mL of the chitosan solution under stirring at 100 rpm. The obtained solution was poured in Petri dishes (47 mm in diameter), followed by oven drying at 40 °C overnight. Then, 1M sodium hydroxide was added to the obtained films to neutralize any residual acid followed by washing with distilled water until constant pH. The films were dried at room temperature for 24 h and stored in desiccator until use. 

### 2.5. Characterization of the Films 

#### 2.5.1. FTIR

Infrared spectroscopy analysis was performed using a Prestige-21 Shimadzu IR spectrometer (Tokyo, Japan). Dried MHF and films were placed on steel plates and analyzed directly by attenuated total reflectance (ATR). Analyzes were performed in the 4000–700 cm^−1^ region with 15 scans and spectral resolution of 4 cm^−1^.

#### 2.5.2. XRD

XRD analysis was performed using a Bruker D2 Phaser (Massachusetts, USA) with CuK_α_ radiation (λ = 1.54 Å) at a voltage of 30 kV, a current of 10 mA, and a Lynxeye detector. The samples were scanned at room temperature over a period of 2 h at a range of 5° to 60° (0.05°/s).

#### 2.5.3. TGA

TG thermograms were obtained through a Shimadzu 60AH (Tokyo, Japan) at a temperature range of 25–600 °C using alumina crucibles with approximately 3 mg of samples under dynamic nitrogen atmosphere (50 mL/min) and heating rate of 10 °C/min. Thermogravimetry/derivative thermogravimetry (TG/DTG) was calibrated using the standard CaC_2_O_4_ H_2_O.

#### 2.5.4. DSC

DSC thermal analyzes were performed on a DSC-60A Shimadzu (Tokyo, Japan) using approximately 3 mg of sample sealed in aluminum crucibles, under dynamic nitrogen atmosphere (50 mL/min), heating rate of 10 °C/min, and a temperature range of 25–400 °C. The temperature and heat flow of the DSC instrument were calibrated with indium (melting point = 157.5 °C and ∆H = 26.7 J g^−1^).

#### 2.5.5. SEM

The samples were previously mounted on the specimen holder using double-sided adhesive tapes followed by morphological analysis using an SEM Hitachi (Tokyo, Japan) with magnifications of 100×, 500×, and 1000×. Scanning electron microscopy images were obtained at an acceleration potential of 15 kV under reduced pressure.

#### 2.5.6. AFM

Surface roughness and film morphology were assessed at room temperature using a multimode scanning probe microscope with a Nanoscope III controller (Digital Instruments, Santa Bárbara, CA, USA) operated at intermittent contact mode. The scan size was 1 μm^2^, and the scan rate was 1.97 Hz with 512 pixels collected per line.

#### 2.5.7. Mechanical Properties

The standard method ASTM D5034 used for the measurement of tensile strength (TS) and elongation at break (EB) was performed with a Tensolab 3000 Mesdan (Puegnago del Garda, Italy). Film samples were cut into 5 × 15 cm rectangular strips, and the tensile test was performed at a speed of 300 mm/min under a controlled environment of 21 °C and 65% relative humidity. A stress-strain curve was recorded using a computer. The tensile strength and percentage of elongation at break were calculated using Equation (1) and Equation (2), respectively,

TS = *F*_max_/*A* (1)

EB = Δ*L*/*L*_0_ × 100 (2)

where *F*_max_ is the maximum load (*N*), *A* is the initial cross-sectional area (m^2^), Δ*L* is the extension of film strips (m) and *L_0_* is the initial length (m).

#### 2.5.8. Films Thickness

The film thickness was determined by SEM using the method described in Section 2.5.5. The thickness of each sample was measured by taking the SEM image of the cross-section. The thickness was measured at nine random points and reported as the average.

### 2.6. In Vivo Wound Healing Study

#### 2.6.1. Animals

Forty-five female Swiss mice (*Mus musculus*) between six and eight weeks of age (25–30 g) were housed in the UFRN Health Sciences Center (CCS) vivarium under controlled lighting (12 h light/dark cycle) and temperature (23 ± 2 °C). The animals received water and commercial food (Presence^®^, Agroline, Campo Grande, MS, Brazil) ad libitum. This study was previously approved by the Animal Use Ethics Committee (CEUA) of the Federal University of Rio Grande do Norte (088.007/2018) on 27 March 2018. All experimental procedures were performed in accordance with the National Institutes of Health Guide for the Care and Use of Laboratory Animals (NIH Publications No. 8023, revised 1978). All efforts were made to minimize suffering, and only the minimum number of animals required to produce reliable scientific data was used.

#### 2.6.2. Wound Healing Activity

The animals were randomly divided into three groups (*n* = 15 per group). Group 1 was treated with blank chitosan films (BF), group 2 was treated with chitosan films containing MHF (CMHF), and group 3 was not treated (untreated group). All animals were kept in individual cages until the end of the experiment.

Prior to inflicting the wounds, the animals were submitted to intraperitoneal anesthesia with ketamine and xylazine (100 mg/kg and 10 mg/kg, respectively) and placed in the prone position for shaving the back with a razor blade. After asepsis with 70% alcohol, excisional skin wounds were made in duplicate by pressing the skin of the dorsal region of each animal with a 5 mm diameter circular biopsy punch followed by scissor cutting.

The films were applied to the wounds immediately after their infliction and reapplied when necessary until the animals were euthanized. The injured area was photographed, and its dimension was measured using a digital caliper and Image J software (National Institute of Health, Bethesda, MD) at 0, 2, 5, 7, 10, and 14 days of treatment [25]. Results were expressed as percentage wound closure relative to the original wound size [26] using Equation (3),

% wound closure = wound area day 0 − wound area day n/wound area day 0(3)

where wound area at day 0 was the original wound area after the surgery and wound area at day n was the wound area on n days of post wounding (day 2, 5, 7, 10, and 14).

At the end of each period (day 2, 7, and 14), five animals from each group were euthanized with an overdose of ketamine and xylazine, and a biopsy of the wound was taken for subsequent histological and cytokine analysis.

##### Histological Analysis

The wound biopsy specimens were fixed in 10% buffered formalin, dehydrated, and paraffin embedded. Then, 5-μm-thick samples were obtained for hematoxylin-eosin (H&E) staining and examined by light microscopy (Nikon E200 LED, Minato, Tokyo, Japan). Three sections of the lesions (five animals per group) were analyzed. Morphological changes were investigated using scores whose parameters [27] are shown in Table 1. Masson’s trichrome stained samples were examined by light microscopy at magnifications of 10× and 40×, where 10 different fields were analyzed for collagen fiber deposition near the skin lesion.

##### Determination of Cytokine Concentration

Interleukin 1-β (IL-1β), interleukin 10 (IL-10), and TNF-α levels were measured by the enzyme linked immunosorbent assay (ELISA) using R&D kits (Minneapolis, USA). The wound tissue was homogenized in 1:6 saline phosphate buffer, which was centrifuged at 4285 rpm at 4 °C for 10 min (Novatechnique NT 805, SP, Brazil), and the supernatant was used for absorbance determination (Mindray MR-96A at 450 nm). The concentration of IL-1β and IL-10 was determined according to the kit protocol (detection range 62.5–4000 pg/mL).

### 2.7. Statistical Analysis

Data were presented as mean ± SD (standard deviation). Data were analyzed by analysis of variance (ANOVA) followed by Tukey and Bonferroni test using GraphPad Prism software (San Diego, CA, USA). Values of *p* < 0.05 were considered statistically significant.

## 3. Results and Discussion

### 3.1. Chemical Composition of MHF

**Figure 1:**
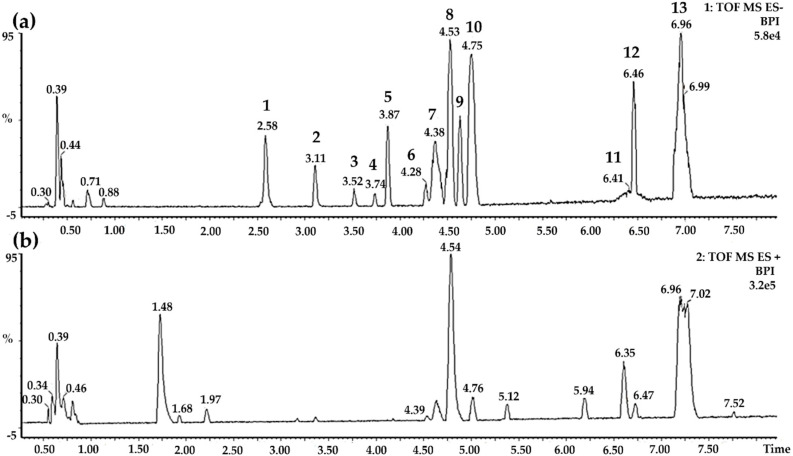
Chemical composition of *Mansoa hirsuta* fraction (MHF). (**a**) Electrospray ionization (ESI) base peak ion (BPI) chromatogram of the *M. hirsuta* fraction analyzed by ultra-high performance liquid chromatography-quadrupole time-of-flight mass spectrometry (UPLC-QTOF-MS-ES-); (**b**) ESI base peak ion (BPI) chromatogram of the *M. hirsuta* fraction analyzed by UPLC-QTOF-MS-ES+.

The *M. hirsuta* fraction was subjected to UPLC-QTOF-MS/MS for chemical profiling and structural characterization. Thirteen acid triterpenes were characterized (Figure 1). Table 2 summarizes the most common ion products observed in the MS/MS spectra. The compounds presented unsaturation, hydroxyls and additional coumaroyl groups in their structures. The mass spectra interpretation of MHF indicates that this fraction is rich in acid triterpenes that can be derived from oleanolic and ursolic acids. The neutral losses of H_2_O, HCOOH, and CH_4_ are observed from all structures. The loss of H_2_O is characteristic of an OH group, whereas the loss of HCOOH is a characteristic of fragmentation of pentacyclic triterpenes [28,29].

Pentacyclic triterpenes are secondary metabolites widely found in a variety of organisms such as bacteria, fungi, plants, and mammals [30]. These natural compounds are attracting interest due to their important pharmacological activities including antitumor, antibacterial, antiviral, anti-inflammatory, anti-diabetic and immunomodulatory [31,32]. Thus, *M. hirsuta* fraction, enriched in pentacyclic triterpenes, might be a promising potential source for the development of new wound dressings. In fact, studies have already reported beneficial effects of triterpenes on wound healing by inducing basal cell proliferation, keratinocyte differentiation and stimulating angiogenesis, and collagen production by fibroblasts [33,34]. These findings provided the basis for the formulation of CMHF.

### 3.2. Preparation and Characterization of the Films

#### 3.2.1. Preparation of Chitosan/*M. hirsuta* Fraction Films

The films were successfully developed using the solvent casting technique where the initial macroscopic evaluation showed that BF films were transparent, whereas CMHFs were light yellow due to the pigments of the *M. hirsuta* fraction. The ethanolic solution of MHF was freely miscible with the chitosan solution, which might have contributed to its homogeneous distribution in the chitosan matrix.

#### 3.2.2. FTIR

**Figure 2:**
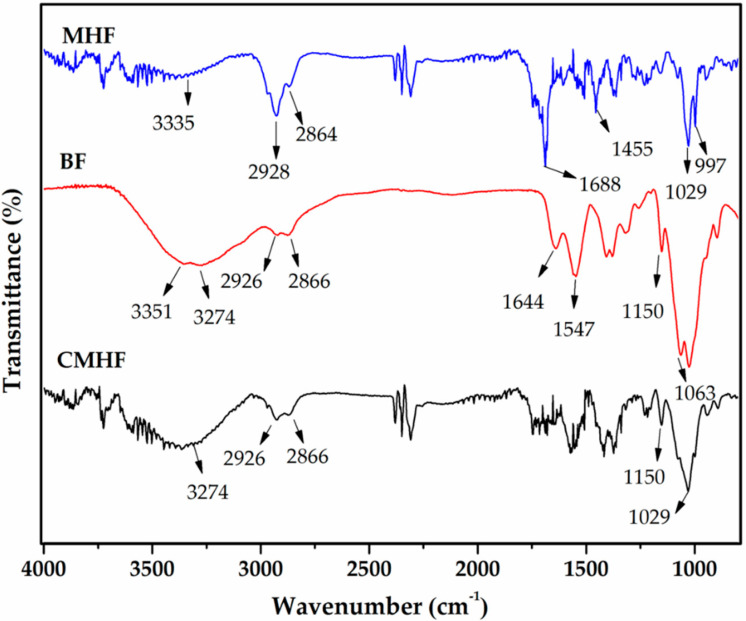
Fourier transform infrared spectroscopy (FTIR) analyses of MHF, blank chitosan film (BF), and chitosan films containing MHF (CMHF).

FTIR analyses were performed with the purpose of elucidating intermolecular interactions between chitosan and the components of the MHF. Therefore, FTIR analyses were carried out on MHF alone, as well as that incorporated into chitosan films. In addition, analyses were performed on chitosan films without MHF (BF) for comparison purposes. The infrared spectrum of MHF (Figure 2 and Figure S1) shows bands characteristic (Table 3) of triterpenoids [35,36,37,38]. For the BF, the spectrum showed the characteristics absorption bands of chitosan as previously reported [39,40,41].

As depicted in Table 3, the FTIR spectrum of CMHF (Figure 2 and Figure S1) show the bands attributed to chitosan (3274, 2926, 2866, 1150 cm^−1^) and MHF (1688, 1455, 1029 e 997 cm^−1^). In addition, the incorporation of MHF into chitosan film resulted in some changes in the FTIR spectrum of BF. The characteristic absorption bands of MHF at 1688, 1455, 1029, and 997 cm^−1^ became less intense because of the dilution of MHF. As the concentration of MHF decreases, the intensities of its characteristic bands might be lower [41]. The absorption band at 1063 cm^−1^ assigned to chitosan was displaced by 1075 cm^−1^ because MHF has a broad peak in this region, and the broad signals of BF and MHF have overlapped. In addition, the intensity of the absorption bands at 1150 and 895 cm^−1^ decreased. Therefore, the FTIR spectrum of CMHF exhibited characteristic absorption bands of both chitosan and MHF, which seems to indicate that only physical interactions took place between them, as no additional peaks or significant changes were observed in the wavelengths of MHF and BF [40].

#### 3.2.3. XRD

**Figure 3:**
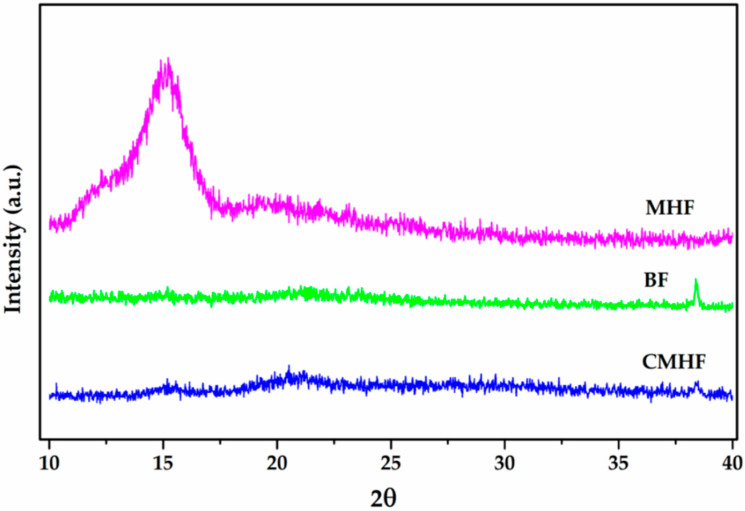
X-ray diffraction (XRD) diffractograms of MHF, BF, and CMHF.

XRD technique was used to evaluate the crystalline or amorphous profile of the films. X-ray diffractograms of MHF, BF, and CMHF are shown in Figure 3. MHF exhibited a predominant amorphous character with diffraction peak around 2θ = 15.0°. BF also showed an amorphous character with a weak diffraction peak around 2θ = 38.0°. With the incorporation of MHF into chitosan film the peaks of MHF and chitosan disappeared and no diffraction peaks were observed for CMHF [42]. The amorphous nature of CMHF is an indication of good miscibility between the components [43].

#### 3.2.4. Thermal Analysis

**Figure 4:**
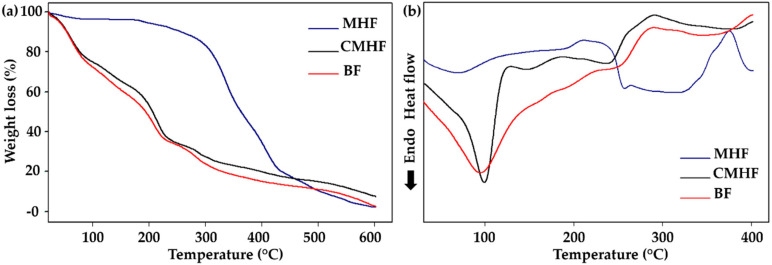
Thermal analysis. (**a**) Thermogravimetry (TGA) thermograms of MHF, BF, and CMHF; (**b**) Differential scanning calorimetry (DSC) thermograms of MHF, BF, and CMHF.

The effect of MHF addition on thermal stability of films was also investigated by TGA with the programmed temperature control for obtaining stability information, as well as for predicting their shelf lives and suifigurstorage conditions [44,45]. According to Figure 4a and Table 4, all samples showed mass losses with increasing temperature. The films exhibited similar thermal degradation with initial decomposition due to the loss of water and acetic acid [46], followed by structural degradation of chitosan and MHF components in later stages [47]. These results indicate that the incorporation of MHF did not change the thermal stability of BF. In the case of MHF, the initial small mass loss of 3% might be due to the loss of water, with thermal degradation in the second and third stages.

Thermal stability of the films was also evaluated by DSC. BF and CMHF (Figure 4b) showed endothermic peaks at 94 °C (BF) and 99 °C (CMHF), which correspond to the energy required for the evaporation of the water present in the samples [48]. The exothermic peak at 290 °C observed in the DSC curve for the films was attributed to the thermal decomposition of chitosan and *M. hirsuta* fraction [49]. An endothermic peak lower than 69 °C was observed in the MHF thermogram due to water loss, followed by an endothermic peak (257 °C) and an exothermic peak (375 °C) corresponding to the degradation of MHF compounds.

The results of DSC analysis were consistent with the results of TGA, indicating that the thermal stability of chitosan films was not significantly affected by the incorporation of MHF. Furthermore, it is important to note that films were thermally stable up to a temperature around 250 °C, indicating that the chemical structure of CMHF was not degraded during manufacturing and storage.

#### 3.2.5. SEM and AFM

**Figure 5:**
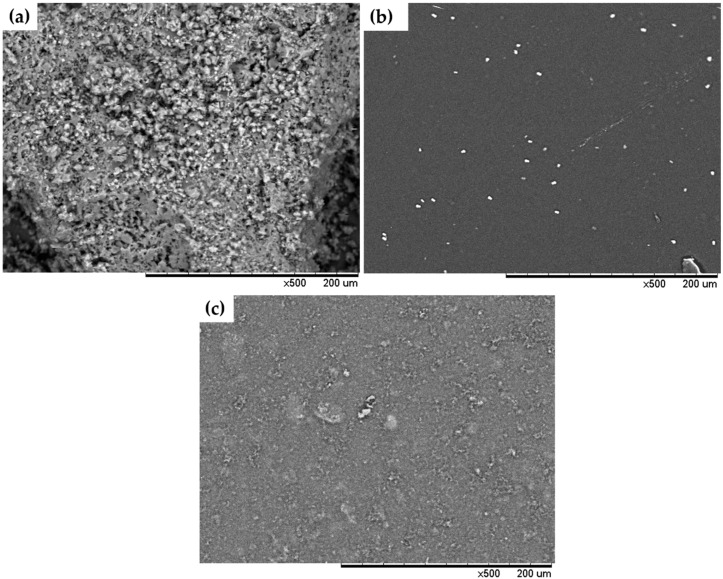
Scanning electron microscopy (SEM) of MHF and films. (**a**) SEM images of MHF; (**b**) SEM images of BF; (**c**) SEM images of CMHF.

Considering that the permeability of the films might be influenced by parameters such as structure, morphology, and homogeneity of the matrix, scanning electron microscopy was used to investigate the microstructure of the films and the distribution of their components [50]. The morphology of MHF was irregular (Figure 5a). The surface structure of BF (Figure 5b) was smooth and continuous, without the presence of microfractures, indicating a compact arrangement of polymer chains [51]. Figure 5c show a high degree of dispersion of the MHF in the chitosan matrix, resulting in changes on the surface’s microstructure of the films, which appear as rough structures with irregular morphology.

**Figure 6:**
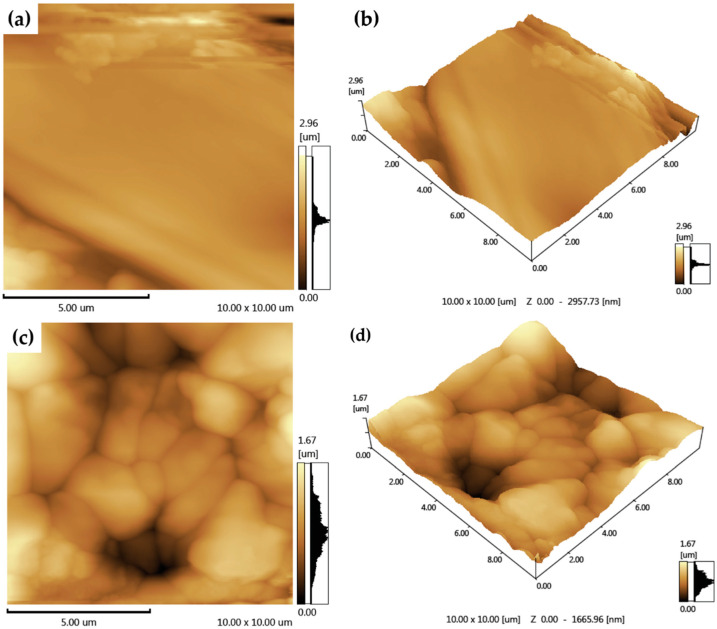
Two-dimensional and 3D topographic images of the films. (**a**,**b**) Two-dimensional and 3D atomic force microscopy (AFM) images of BF; (**c**,**d**) two-dimensional and 3D AFM images of CMHF.

Furthermore, the atomic force microscopy technique was used to further investigate the surface microstructure of the films. The 2D and 3D topographic images are shown in Figure 6. As observed in Figure 6a,b, chitosan film presents a relatively smooth and continuous surface. In contrast, the films containing MHF (Figure 6c,d) showed an uneven and rough structure, which was in accordance with the SEM findings. 

Such findings are particularly interesting as optical, mechanical, adhesive, electrical and various other physicochemical properties of films can be further altered with the changes in the surface morphology [52]. In fact, a rough surface may have benefits for skin regeneration, as it might increase the film’s adhesion capacity and thus promote a faster wound healing process [53,54].

#### 3.2.6. Mechanical Properties and Film Thickness

A wound dressing is usually wrapped around the skin during its application, and when stretched out, it should not rupture. Therefore, it must maintain intact during the entire wound healing time [1,55]. In this current study, the mechanical properties of the films were investigated, and the results are summarized on Table 5. The analysis of data evidenced that the tensile strength of CMHF increased significantly (22.60 ± 2.79) compared to that of BF (9.19 ± 0.78). The improved mechanical performance might be due to the plasticizing effect of MHF [56], which might have increased the mobility of the polymer chains. In fact, a more stretchable and flexible film is important for keeping the integrity of the wound dressing [1,57]. Besides, CMHF exhibited sufficient strength (≥10 MPa) to adapt to skin contours, which would make it easy to apply over the wound [54].

The thickness also affects the properties of wound dressings. Although the thickness of CMHF (Table 5) was low (26.57 ± 2.052), a thin film offers several advantages, including a faster onset of drug action, a reduction in the dose frequency, an enhancement in the drug efficacy, and a more convenient administration through non-invasive routes [58].

### 3.3. Wound Healing Activity

**Figure 7:**
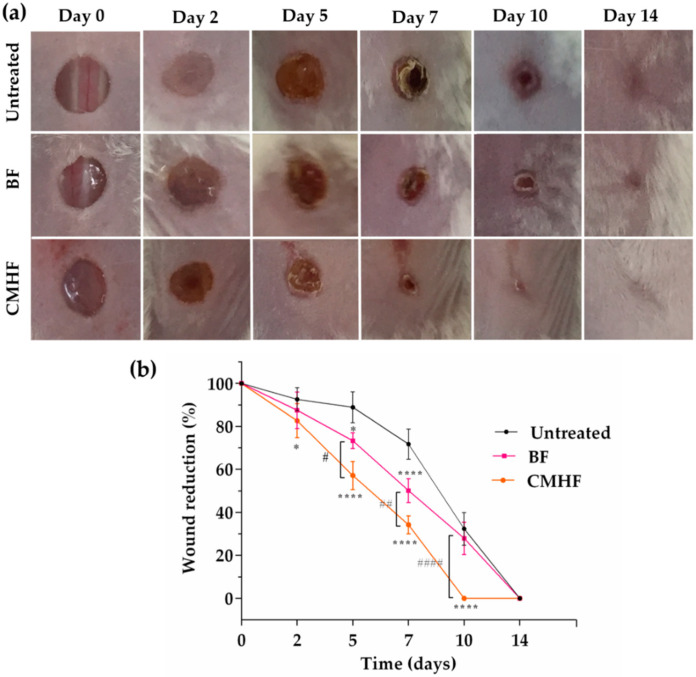
In vivo wound healing study performed on days 0, 2, 5, 7, 10, and 14. (**a**) Representative images of the wound healing process of the untreated, BF, and CMHF groups. (**b**) Wound closure rate measured on days 2, 5, 7, 10, and 14 after treatment with BF and CMHF. Data are expressed as percentage of wound size reduction in comparison with the original wound (day zero). **** *p* < 0.0001 and * *p* < 0.05 compared to untreated group and #### *p* < 0.0001, ## *p* < 0.01, and # *p* < 0.05 compared to BF group using ANOVA followed by Tukey test (*n* = 5).

Considering that the film development was successful and MHF has promising pharmacological properties, the wound healing efficacy of CMHF film was investigated in mice as shown in Figure 7a,b. The percentage of wound closure gradually increased over time (Figure 7b). The CMHF-treated group achieved 40, 62, and 100% of wound contraction after 5, 7, and 10 days of treatment, respectively, which were statistically higher than those of the untreated group (10, 29, and 66%). The BF-treated group reached 25%, 40%, and 69% on the respective days, showing a significant difference from the untreated group only on the fifth and seventh days. The wounds treated with CMHF showed intense formation of granulation tissue on the fifth day of treatment and by the tenth day the wounds were completely closed (Figure 7a). These data indicate that the addition of *M. hirsuta* fraction improved the wound healing effect of chitosan films.

The faster healing of the wounds treated with CMHF may be due to the compounds present in the MHF such as the terpenoids oleanolic acid, and ursolic acid. These triterpenes help to improve wound contraction and facilitate epithelialization. In fact, ursolic acid is reported to stimulate healing by inducing keratinocyte proliferation [59]. In addition, chitosan undergoes gradual depolymerization in vivo by releasing N-acetylglucosamine units, which induces fibroblast proliferation, collagen deposition, and higher levels of hyaluronic acid at the wound site [60,61,62].

#### 3.3.1. Histological Analysis

**Figure 8:**
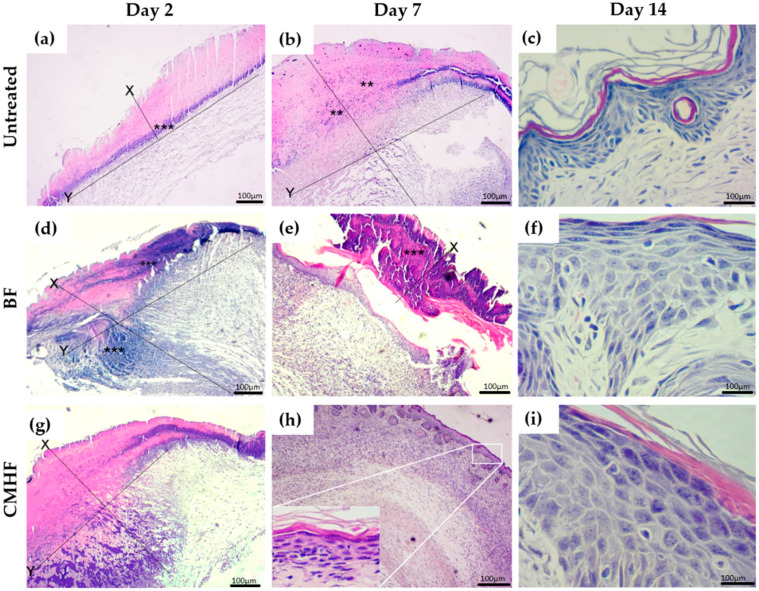
Hematoxylin-eosin (H&E) staining photomicrographs of biopsies of the untreated, BF and CMHF groups assessed at (**a**,**d**,**g**) day 2, (**b**,**e**,**h**) day 7, and (**c**,**f**,**i**) day 14. The x axis indicates lesion depth extending from the epidermis to the dermis and the y axis indicates lesion extension. Statistical difference demonstrated by ANOVA analysis with *p* < 0.05 followed by Bonferroni post-test (*n* = 6).

Histological analysis of the wound tissue from the untreated group as well as BF and CMHF treated groups are depicted in Figure 8. Right after wound infliction, the epithelial cells were totally damaged. Two days later, the untreated (Figure 8a) and BF (Figure 8d) groups presented an area of epithelial discontinuity as well as a purulent fibrin exudate in the underlying area of the connective tissue, which are compatible with ulceration and intense mononuclear inflammatory infiltrate (***). The wound treated with CMHF (Figure 8g) presented an ulceration area with the presence of crust and purulent fibrin exudate. Besides, inflammatory infiltrate in the underlying area of the connective tissue is also observed (***). Seven days after wound infliction, the untreated group (Figure 8b) had a large area with perilesional edema and mononuclear or mixed inflammatory infiltrate. In the group treated with BF (Figure 8e), there is an ulceration area with the presence of crust and focal areas of inflammatory infiltrate. However, the wounds treated with CMHF for seven days show a characteristic area of advanced healing and re-eptellization. In addition, numerous vascular shoots with few cell layers as well as the formation of a thin keratin layer are observed, as shown in Figure 8h. After 14 days, the untreated (Figure 8c) and BF (Figure 8f) groups presented advanced healing processes, whereas the group treated with CMHF showed a completely re-epithelized area (Figure 8i).

**Figure 9:**
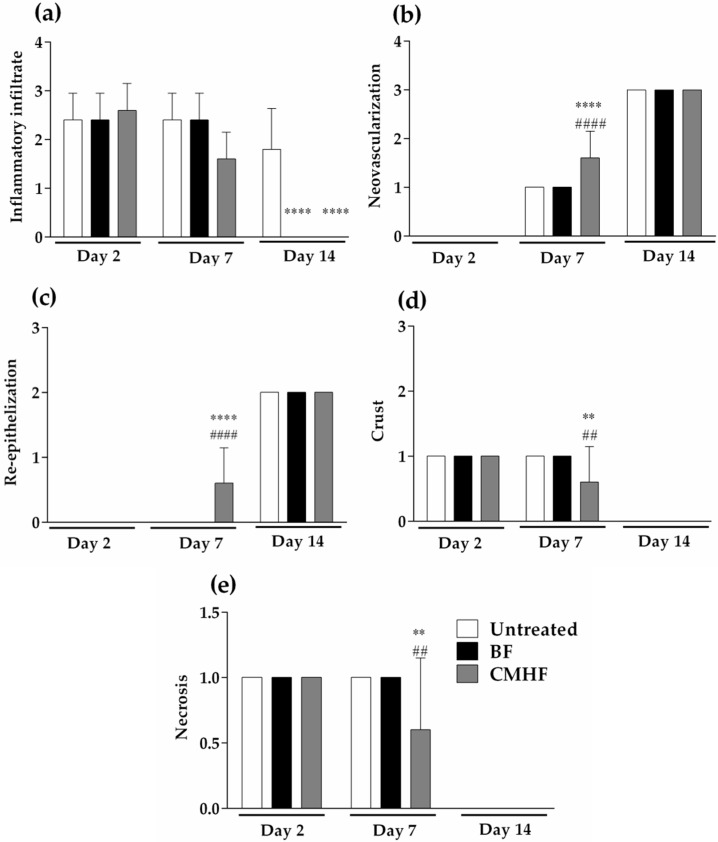
Hystomorphology of H&E-stained epithelial tissue from the untreated, BF, and CMHF groups. (**a**) Inflammatory infiltrate; (**b**) neovascularization; (**c**) re-epithelization; (**d**) crust; (**e**) necrosis. Statistical difference demonstrated by ANOVA analysis followed by Tukey post-test. Data represents the mean of the values obtained from five animals and the vertical lines indicate the standard deviation. **** *p* < 0.0001, and ** *p* < 0.01 for comparison between the untreated and BF groups; #### *p* < 0.001, and ## *p* < 0.01 for comparison between the BF and CMHF groups.

Morphological changes were also analyzed using scores (Figure 9). The inflammatory infiltrate (Figure 9a) on the group treated with CMHF declined after seven days of treatment, showing a total reduction on day 14 compared with the untreated group which still had some inflamed areas. However, after seven days of treatment with CMHF, an increase (53%) in neovascularization (Figure 9b) was observed with complete re-epithelization (Figure 9c) taking place on day 14. In addition, a significant decrease (60%) in the crust (Figure 9d) and necrosis (Figure 9e) after seven days of treatment with CMHF was observed in comparison with the untreated and BF groups.

**Figure 10:**
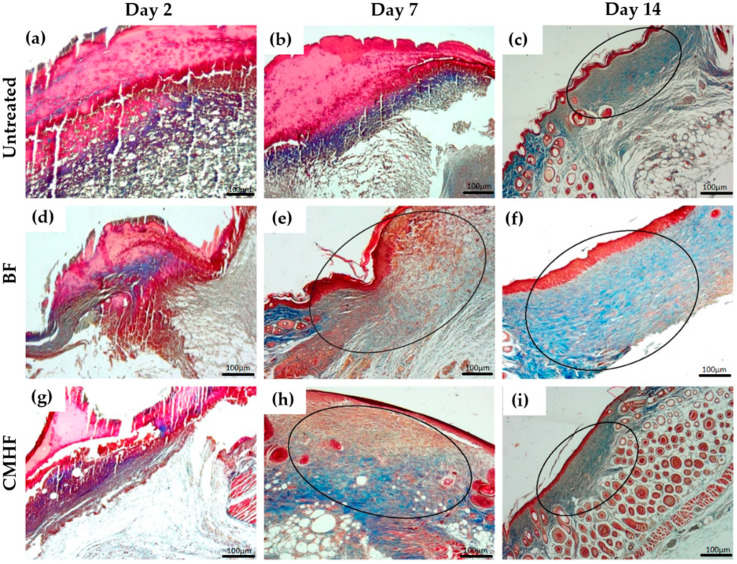
Photomicrographs of Masson’s trichrome-stained tissue fragments from the untreated, BF, and CMHF, where collagen deposition was assessed (**a**,**d**,**g**) two days after wound infliction, (**b**,**e**,**h**) seven days after wound infliction, and (**c**,**f**,**i**) 14 days after wound infliction. Ten different fields were observed with magnifications of 10× and 40× near the skin lesion for collagen fiber deposition at different healing stages.

Masson’s trichrome staining was used for the analysis of collagen fibers in the wound area on days 2, 7, and 14 (Figure 10). Collagen deposition was scarce in all groups (Figure 10a,d,g) on day 2, as the wounds were in the early stage of the healing process. After seven days (Figure 10b,e,h), the wounds treated with CMHF showed a more intense blue color, indicating higher collagen deposition compared to the other groups where a very low deposition of collagen was observed. Figure 10c,f,i shows the final stage of healing, indicating that all groups had highly mature collagen fibers at day 14.

These results demonstrated that treatment with CMHF was effective in enhancing wound healing as it promoted resolution of inflammation, which is essential for efficient healing. In addition, it significantly increased re-epithelialization and neovascularization, which were evidenced by higher keratinocyte proliferation as well as higher branching and extension of adjacent blood vessels [63,64]. Furthermore, animals treated with CMHF for seven days had lower necrosis and crust with increased collagen fiber deposit. These findings demonstrate that the incorporation of *M. hirsuta* fraction into chitosan film improved the wound healing process.

#### 3.3.2. Cytokine Analysis

Cytokines such as TNF-α, IL-1β, and IL-10 are involved in the wound healing process [65]. During the onset of the inflammatory phase, activated macrophages produce IL-1β and TNF-α, which induce inflammatory leukocyte recruitment into the wounded tissues and stimulate the activity of fibroblasts as well as the synthesis and breakdown of extracellular matrix proteins that are involved in the healing of the injured tissues [66]. IL-10 is a cytokine that acts during the resolution phase of inflammation, regulating the expression of pro-inflammatory cytokines that reduce tissue damage [67]. This process facilitates wound healing in lesions caused by infection or inflammation [67].

**Figure 11:**
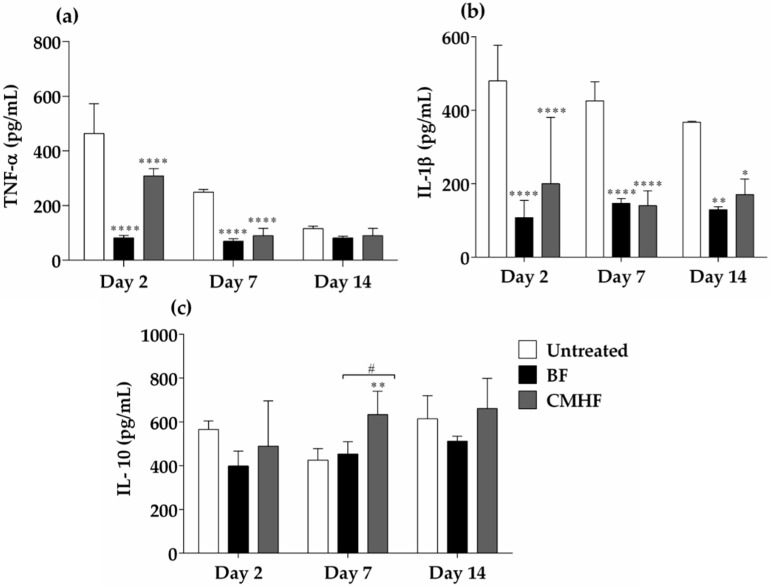
Cytokine analysis. (**a**) Cytokine TNF-α expressed on the untreated, BF, and CMHF groups using an enzyme-linked immunosorbent assay. (**b**) Cytokine IL-1β expressed on the untreated, BF, and CMHF groups using an enzyme-linked immunosorbent assay. (**c**) Cytokine IL-10 expressed on the untreated, BF, and CMHF groups using an enzyme-linked immunosorbent assay. Statistical difference demonstrated by ANOVA analysis followed by Tukey post-test. Data represents the mean of the values obtained from three animals and the vertical lines indicate the standard deviation. **** *p* < 0.0001, ** *p* < 0.01, and * *p* < 0.05, for comparison between the untreated and BF groups; # *p* < 0.05 for comparison between the BF and CMHF groups.

In this study, the levels of TNF-α (Figure 11a), IL-1β (Figure 11b), and IL-10 (Figure 11c) were evaluated 14 days after wound infliction. Both BF and CMHF were found to significantly decrease TNF-α and IL-1β levels. On the other hand, IL-10 showed higher expression in the group treated with CMHF when compared with the untreated and BF groups. These results show that CMHF effectively decreased the inflammatory response and accelerated the healing process.

## 4. Conclusions

The results of this study showed that a novel chitosan film entrapped with *Mansoa hirsuta* fraction was successfully developed for wound healing application. The mass spectra interpretation of MHF indicates that this fraction is rich in acid triterpenes that can be derived from oleanolic and ursolic acids, which are beneficial for human health due to their various pharmacological activities including anti-inflammatory and immunomodulatory. FTIR spectrum of CMHF exhibited characteristic bands of both chitosan and MHF revealing a mixture of both. In addition, the films presented an amorphous nature, a reasonable thermostability and a homogeneous dispersion of MHF in the chitosan matrix, resulting in a rough structure with high stretching ability. Such properties might have helped CMHF to improve wound contraction and to facilitate epithelialization. Indeed, these films showed potential to be used as a novel wound dressing as it effectively accelerated the healing stages by increasing wound closure rate, with 100% contraction after only 10 days of treatment. Therefore, the chitosan film with *M. hirsuta* fraction is a promising dressing for stimulating wound repair and regeneration.

## Figures and Tables

**Table 1:** Criteria for histological analysis of healing.

Scores	Inflammatory Infiltrate	Neovascularization	Reepithelization	Granulation	Crust and Necrosis
0	Absent	Absent	Absent	Absent	Absent
1	Discrete	Initial	Partial	Present	Present
2	Moderate	Partial	Complete	-	-
3	Intense	Complete	-	-	-

**Table 2:** Characterization of acid triterpenes of *M. hirsuta* fraction by UPLC/QTOF-MS-MS.

Peak	*t*R (min)	[M-H]^−^	[M-H]^−^ Calculated	[M-H]^+^	[M-H]^+^ Calculated	MS^2^ Ions (ESI Positive)	Identification
1	2.57	487.3426	487.3428	489.3581	489.3574	471.3492 [M+H-H_2_O]^+^, 453.3381 [M+H-2H_2_O]^+^, 435.3277 [M+H-3H_2_O]^+^, 407.3332 [M+H-2H_2_O-HCOOH]^+^, 325.6983 [M+H-3H_2_O-HCOOH-4CH_4_]^+^	Trihydroxyolean-en-oic acid or trihydroxyurs-en-oic acid
2	3.09	487.3426	487.3428	489.3581	489.3574	471.3492 [M+H-H_2_O]^+^, 453.3381 [M+H-2H_2_O]^+^, 435.3277 [M+H-3H_2_O]^+^, 407.3332 [M+H-2H_2_O-HCOOH]^+^, 325.6983 [M+H-3H_2_O-HCOOH-4CH_4_]^+^	Trihydroxyolean-en-oic acid or trihydroxyurs-en-oic acid (isomer)
3	3.52	487.3426	487.3428	489.3577	489.3574	471.3492 [M+H-H_2_O]^+^, 453.3381 [M+H-2H_2_O]^+^, 435.3277 [M+H-3H_2_O]^+^, 407.3332 [M+H-2H_2_O-HCOOH]^+^, 325.6983 [M+H-3H_2_O-HCOOH-4CH_4_]^+^	Trihydroxyolean-en-oic acid or trihydroxyurs-en-oic acid (isomer)
4	3.72	485.3273	485.3272	487.3428	487.3418	469.3329 [M+H-H_2_O]^+^, 451.3232 [M+H-2H_2_O]^+^, 423.3286 [M+H-H_2_O-HCOOH]^+^, 405.3201 [M+H-2H_2_O-HCOOH]^+^, 324.6904 [M+H-3H_2_O-HCOOH-4CH_4_]^+^	Trihydroxyurs-dien-oic acid or trihydroxyolean-dien-oic acid
5	3.86	471.3479	471.3479	473.3634	473.3625	455.3531 [M+H-H_2_O]^+^, 437.3423 [M+H-2H_2_O]^+^, 409.3479 [M+H-H_2_O-HCOOH]^+^, 391.3400 [M+H-2H_2_O-HCOOH]^+^	Dihydroxyurs-en-oic acid or dihydroxyolean-dien-oic acid
6	4.25	471.3479	471.3479	473.3627	473.3625	455.3531 [M+H-H_2_O]^+^, 437.3423 [M+H-2H_2_O]^+^, 409.3479 [M+H-H_2_O-HCOOH]^+^, 391.3400 [M+H-2H_2_O-HCOOH]^+^	Dihydroxyurs-en-oic acid or dihydroxyolean-dien-oic acid (isomer)
7	4.36	471.3479	471.3479	473.3630	473.3625	455.3531 [M+H-H_2_O]^+^, 437.3423 [M+H-2H_2_O]^+^, 409.3479 [M+H-H_2_O-HCOOH]^+^, 391.3400 [M+H-2H_2_O-HCOOH]^+^	Dihydroxyurs-en-oic acid or dihydroxyolean-dien-oic acid (isomer)
8	4.52	469.3323	469.3323	471.3468	471.3468	453.3372 [M+H-H_2_O]^+^, 437.3422 [M+H-H_2_O-CH_4_]^+^, 425.2431 [M+H-HCOOH]^+^, 407.3314 [M+H-H_2_O-HCOOH]^+^, 325.6804 [M+H-2H_2_O-HCOOH-4CH_4_]^+^	Hydroxy-oxoleana-en-oic acid or hydroxy-oxours-en-oic acid
9	4.62	471.3479	471.3479	473.3629	473.3625	455.3531 [M+H-H_2_O]^+^, 437.3423 [M+H-2H_2_O]^+^, 409.3479 [M+H-H_2_O-HCOOH]^+^, 391.3400 [M+H-2H_2_O-HCOOH]^+^	Dihydroxyurs-en-oic acid or dihydroxyolean-dien-oic acid (isomer)
10	4.74	471.3479	471.3479	473.3631	473.3625	455.3531 [M+H-H_2_O]^+^, 437.3423 [M+H-2H_2_O]^+^, 409.3479 [M+H-H_2_O-HCOOH]^+^, 391.3400 [M+H-2H_2_O-HCOOH]^+^	Dihydroxyurs-en-oic acid or dihydroxyolean-dien-oic acid (isomer)
11	5.58	471.3479	471.3479	473.3636	473.3625	455.3531 [M+H-H_2_O]^+^, 437.3423 [M+H-2H_2_O]^+^, 409.3479 [M+H-H_2_O-HCOOH]^+^, 391.3400 [M+H-2H_2_O-HCOOH]^+^	Dihydroxyurs-en-oic acid or dihydroxyolean-dien-oic acid (isomer)
12	6.45	617.3858	617.3847	619.3985	619.3993	455.3524 [M+H-coumaroyl]^+^, 437.3419 [M+H-coumaroyl-H_2_O]^+^, 409.3480 [M+H-coumaroyl-HCOOH]^+^	Coumaroyl-hydroxy-urs-en-oic acid
13	6.95	455.3534	455.3530	457.3679	457.3673	439.3578 [M+H-H_2_O]^+^, 411.3625 [M+H-HCOOH]^+^, 393.3523 [M+H-HCOOH-H_2_O]^+^	Ursolic acid or oleanolic acid

**Table 3:** FTIR analysis of MHF, BF, and CHMF.

Samples	Wavenumber (cm^−1^)	Functional Groups and Types of Vibration	References
MHF	3335 cm^−1^	OH– stretching	[37]
	2928 cm^−1^	CH_3_– (aliphatic) asymmetric stretching vibration	[36]
	2864 cm^−1^	CH_3_– (aliphatic) symmetric stretching	[36]
	1688 cm^−1^	C=O stretching vibration	[36]
	1455 cm^−1^	Angular deformation vibration of CH alkenes	[38]
	1029 and 997 cm^−1^	Symmetric C–O stretches and olefinic groups	[36]
BF	3351 cm^−1^ and 3274 cm^−1^	OH– stretching which overlaps with NH–stretching	[40]
	2926 cm^−1^; 2866 cm^−1^	CH_2_–; CH– stretching vibrations	[41]
	1644 cm^−1^; 1547 cm^−1^	C=O stretching (amide I); NH–bending (amide II)	[39]
	1377 cm^−1^	Acetamide groups	[39]
	1150 cm^−1^	Anti-symmetric stretching of the C–O–C bridge	[41]
	1063 cm^−1^; 890 cm^−1^	Skeletal vibrations involving the C–O stretching; vibration of C–C skeleton	[41]
CMHF	3274 cm^−1^	NH–stretching	[40]
	2926 cm^−1^; 2866 cm^−1^	CH_2_–; CH– stretching vibrations	[41]
	1688 cm^−1^	C=O stretching vibration	[36]
	1455 cm^−1^	Angular deformation vibration of CH alkenes	[38]
	1372 cm^−1^	Acetamide groups	[39]
	1150 cm^−1^	Anti-symmetric stretching of the C–O–C bridge	[41]
	1075 cm^−1^	Skeletal vibrations involving the C–O stretching;	[41]
	1029 and 997 cm^−1^	Symmetric C–O stretches and olefinic groups	[36]
	890 cm^−1^	Vibration of C–C skeleton	[41]

**Table 4:** The thermal behavior of MHF and films determined by TGA.

Samples	First Stage			Seconde Stage			Third Stage		
	Start (°C)	End (°C)	Wt. loss (%)	Start (°C)	End (°C)	Wt. Loss (%)	Start (°C)	End (°C)	Wt. Loss (%)
MHF	20	75	3.00	313	356	25.84	382	435	22.35
BF	44	109	23.88	191	262	19.41	277	370	11.90
CMHF	38	100	21.57	186	251	24.47	280	349	8.00

**Table 5:** Stress, elongation at break, and average break time of BF and CMHF films.

Sample	Tensile Strength (Mpa)	Elongation at Break (%)	Thickness (μm)
BF	9.19 ± 0.78	51.86 ± 10.80	57.89 ± 4.328
CMHF	22.60 ± 2.79 *	68.75 ± 6.11	26.57 ± 2.052 *

* *p* < 0.05 compared to BF.

## Supplementary Materials

The following are available online at https://www.mdpi.com/1999-4923/12/6/484/s1, Figure S1: FTIR spectra of MHF, BF, and CMHF.
