# Supplementary material for: Chitosan Film Containing Mansoa hirsuta Fraction for Wound Healing
Source: Pharmaceutics. 2020 May 27;12(6):484. doi: 10.3390/pharmaceutics12060484 (PMC7356783; doi:10.3390/pharmaceutics12060484)
Supplement: Supplementary file 1 [file pharmaceutics-12-00484-s001.pdf]

pharmaceutics

Supplementary Materials: Chitosan Film Containing *Mansoa hirsuta* Fraction for Wound Healing

Joquebede Rodrigues Pereira, Gabriela Suassuna Bezerra, Allanny Alves Furtado, Thaís Gomes de Carvalho, Valéria Costa da Silva, Amanda Lins Bispo Monteiro, Gerlane Coelho Bernardo Guerra, Raimundo Fernandes de Araújo Júnior, Antônio Euzébio Goulart Sant'Ana, Matheus de Freitas Fernandes-Pedrosa, Daniel de Melo Silva, Eduardo Pereira de Azevedo, Tania Maria Sarmento Silva, Telma Maria Araújo Moura Lemos and Ádley Antonini Neves de Lima

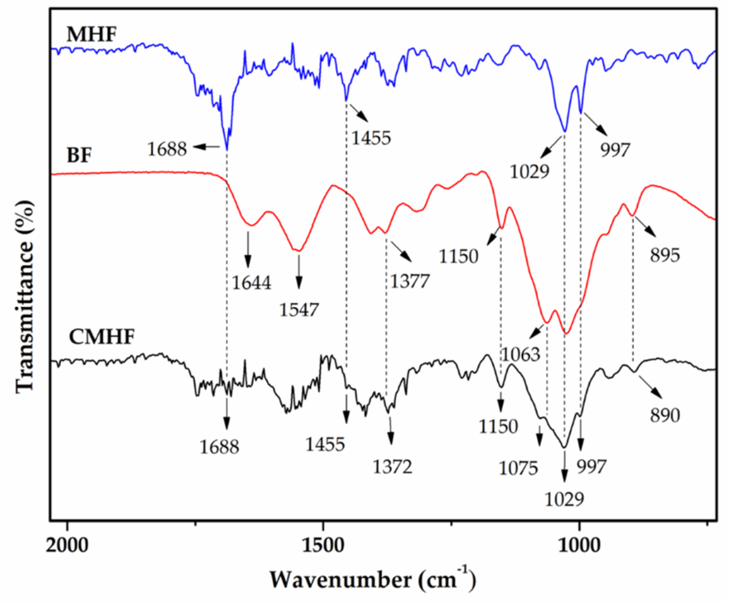

Figure S1. FTIR spectra of MHF, BF, and CMHF.
